# Sacral neuromodulation with ultra-low stimulation intensity is effective in faecal incontinence – results from a randomised study with a one-stage implant procedure

**DOI:** 10.1007/s10151-025-03254-9

**Published:** 2025-12-24

**Authors:** J. Duelund-Jakobsen, S. Buntzen, L. Lundby, S. Laurberg, M. Sørensen, M. Rydningen

**Affiliations:** 1 Pelvic Floor Unit, Department of Surgery, Aarhus University Hospital, Palle Juul-Jensens Boulevard 99, 8200 Aarhus N, Denmark; 2 Clinical Department of Medicine, Aarhus University, Aarhus N, Denmark; 3 Department of Gastroenterological Surgery, National Advisory Board of Incontinence and Pelvic Floor Health of Norway, University Hospital of North Norway, Tromsoe, Norway; 4 Department of Clinical Medicine, Arctic University of North Norway, Tromsoe, Norway; 5 Department of Surgical and Medical Gastroenterology, Hvidovre University Hospital, Hvidovre, Denmark

**Keywords:** Faecal incontinence, Sacral neuromodulation, Quality of life, Functional outcome

## Abstract

**Introduction:**

In sacral neuromodulation (SNM), the stimulation intensity is set at the sensory threshold (ST) level. However, subsensory stimulation as low as 50% of ST has proven effective in reducing faecal incontinence episodes.

**Aim:**

To explore the relationship between functional outcomes and varying subsensory stimulation amplitude in newly implanted patients.

**Method:**

This randomised, double-blind study was designed to include patients with ≥ 1 faecal incontinence episodes/week despite maximal conservative therapy. As part of another trial, patients were offered a one-stage procedure. Postoperatively, patients were randomised into two groups. G-1 received stimulation at 0.05 V, at 50% and 90% of the ST in three 4-week periods, followed by 12 weeks of stimulation at the ST. G-2 received stimulation at 90% of the ST in three 4-week periods, followed by 12 weeks of stimulation at ST. Patients were evaluated after each period using St. Marks’s Incontinence Score and a visual analogue scale (VAS) for patient satisfaction regarding social function, bowel function and quality-of-life, along with a bowel habit diary.

**Results:**

In total, 73 patients with a median age of 60 years [interquartile range (IQR: 50–69 years)] completed the trial. Faecal incontinence episodes were significantly reduced at all follow-ups, with no differences between groups. The only statistical difference between groups was deltaVAS for bowel function after 4 weeks. In G-1 with ultra-low stimulation amplitude [0.05 V – equivalent to 9.6% (IQR: 6.5–13.4) of ST], the improvement compared with baseline was 30 points (IQR: 10–50) significantly lower than G-2 with an improvement of 50 points (IQR: 10–70) (*p*-value: 0.05).

**Conclusions:**

Subsensory stimulation is feasible in newly implanted patients with faecal incontinence. An amplitude of 0.05 V is as effective on the functional outcomes as stimulation with higher amplitudes.

## Introduction

Faecal incontinence (FI) has substantial negative implications for the affected individual and their family. Additionally, it is associated with a high level of stigma and has a major negative impact on quality of life [1, 2]. FI affects approximately 16% of the population but may be referred to as a ‘silent affliction’ since the magnitude of the problem is likely to be underestimated because most individuals are reluctant to consult healthcare providers [3, 4].

The latest guideline for management of FI recommends first-line conservative treatment such as fibre supplements, constipating drugs, biofeedback therapy or transanal irrigation [5]. These treatments have been proven effective in approximately 72% of patients attending a specialist nurse-led clinic [6]. If conservative treatment is insufficient to improve continence satisfactorily, sacral neuromodulation (SNM) may be considered [5].

Since its introduction in 1995, SNM has been proven to be an effective long-term minimally invasive treatment [7–9]. SNM is approved as a two-stage procedure. The first stage (stage I) is performed with a temporary or permanent lead connected to an extension wire that is externalised and connected to an extracorporeal neurostimulator. The treatment response is evaluated after 2–3 weeks before the final decision for a complete implant (stage II). Transitioning from stage I to stage II typically requires a ≥ 50% reduction in incontinence episodes or the number of days impacted by incontinence. With the introduction of the standardised implantation technique [10], the number of patients proceeding from stage I (≥ 50% symptom reduction) to stage II is approaching 90% [11, 12]. The stimulation amplitude has been traditionally set at an individual threshold corresponding to the perception of stimulation, known as the sensory threshold (ST). It has been documented in a randomised trial and in following prospective trials that the stimulation amplitude in patients suffering from FI can be set 50% below ST without compromising treatment efficacy [13, 14]. Current evidence regarding subsensory stimulation (below ST) has been demonstrated in patients stimulated for several years at the ST. We currently lack knowledge about subsensory stimulation and its effectiveness on functional outcomes immediately after implantation. Subsensory stimulation offers several advantages. Firstly, it would eliminate the patient’s discomfort associated with stimulation at the ST. Secondly, reducing the energy consumption of the neurostimulator would increase its life expectancy, subsequently lowering treatment costs. Thirdly, the theoretical risk of nerve damage from electrical stimulation is decreased as less energy is delivered to the surrounding tissue.

This randomised, double-blind study was a part of another study [12] designed to investigate the relationship between functional outcomes and different subsensory stimulation amplitudes in de novo-implanted patients with FI.

## Materials and methods

Patients were recruited from three tertiary referral centres for FI treatment: Aarhus University Hospital, Hvidovre University Hospital, Denmark, and the University Hospital of North Norway, Tromsoe. Study inclusion started in November 2016 and ended in November 2019.

Patients diagnosed with idiopathic FI or FI due to an external anal sphincter defect of ≤ 160° were eligible. The integrity of the anal sphincter was assessed using transanal ultrasound. All patients were to report weekly FI after maximal conservative therapy had been attempted. Patients were excluded from the trial if they previously had received electrical stimulation or injection/bulking therapies for FI. A complete list of inclusion and exclusion criteria has been published previously [12].

As part of another trial recently published [12], these patients underwent one-stage implantation, without a prior test (stage I) if they, during the operation, had a motor response (contraction) of the external anal sphincter in three or more poles and at least one pole of the quadripolar leads with a motor threshold ≤ 1.5 mAmp [12]. The permanent lead Model 3889 (Quadripolar lead for electrical stimulation^®^, Medtronic, Minneapolis, MN) and a neurostimulator InterStim-II (Medtronic, Minneapolis, MN) were used. If the patients did not fulfil the perioperative requirement for a one-stage implant, they were excluded from the study and offered a conventional stage-I test. Perioperatively, the amplitude needed to elicit a contraction of the external anal sphincter was evaluated using the Verify^®^ system (Medtronic). The Verify^®^ system delivers stimulation with a constant current technology, and the amplitude is reported in mAmp. The permanent implant (InterStim-II) provides electrical stimulation using a constant voltage basis (volt). Both systems provide the same energy to the nerve as long as the resistance in the electrical circuit remains unchanged [15].

The implantations were performed during deep sedation or general anaesthesia to document the motor response during lead placement. All patients received preoperative intravenous prophylactic antibiotics.

Patients fulfilling the perioperative criteria were randomised on the day after surgery using sealed envelopes into one of two groups (Table 1). Participants and data assessor (J.D.J.) were blinded during the study phase, and only the programming nurses were aware of the actual randomisation group. The ST was measured the first postoperative day and was used as a reference for the following reprogramming sessions. During the first 12 weeks after one-stage implantation, the stimulation amplitude was increased after 4 weeks for patients in group one (G-1) from 0.05 V (lowest possible setting) to 50% and subsequently after 4 weeks to 90% of ST. In group two (G-2), patients were stimulated at 90% of ST during the first 12 weeks; however, they were seen by the programming nurses, and the stimulation status of the neurostimulator was recorded to ensure blinding of the patients. All patients were stimulated at the ST in the following 12 weeks (week 13–24) (Table 1). Thereafter, the study ended, and patients were followed up according to standard practise in each centre.

**Table 1:** Stimulation amplitude during the different trial periods

	Weeks 1–4	Weeks 5–8	Weeks 9–12	Weeks 13–24
Group 1	0.05 volts	50% of ST	90% of ST	ST
Group 2	90% of ST	90% of ST	90% of ST	ST

ST, sensory threshold

At baseline and during each stimulation period, patients were requested to complete a series of questionnaires (How would you rate your overall satisfaction with bowel function, social function and quality of life during the past 4 weeks?), which were assessed using a numerical visual analogue scale (VAS) with a range from 0 to 100, where 100 indicates excellent function and 0 indicates poor function [16], St. Marks Incontinence Score [17]) and a 3-week bowel habit diary.

### Ethics

The protocol for the study was approved by the Regional Committee on Biomedical Research Ethics, Denmark and the Patient Protection Representative Norway (SJO125) and registered at ClinicalTrials.gov (22 August 2017, NCT03261622). Medtronic was notified about the study but did not participate in the planning, completion or reporting. In case of unexpected adverse events related to the study, patients were covered by national healthcare insurance.

#### Statistical analysis

A sample size calculation (based on a non-inferiority study design) was carried out to determine the number of patients needed to demonstrate that a one-stage implantation is non-inferior to a stage I + II procedure [12]. In total, 60 patients were planned for inclusion. To account for dropout and possible infection leading to explanation, the target for inclusion was 75 patients. Each department could include a maximum of 30 patients. A sample size calculation or power calculation was not performed specifically for the randomised part of the trial. Baseline data are presented as median and interquartile range (IQR) or mean and standard deviation (SD). Data obtained during follow-up are presented as delta values (baseline versus specific period) for enhanced visualisation of differences. Wilcoxon signed-rank test was used to compare results obtained at the initiation of this study and follow-up at 4, 8 and 12 weeks between the two randomisation groups. A paired *t*-test was used to compare the baseline and 24-week follow-ups. In case of missing values, the result of that specific question/score was discharged at the specific follow-up. Values of *p* ≤ 0.05 were considered statistically significant. All statistical analyses were conducted using Stata version 10.1. (Stata Corporation, 4905 Lakeway Drive, TX 77846, USA).

## Results

Overall, 85 patients were eligible, of whom 76 (72 female) gave written consent to participate. Three patients were excluded during the trial. One patient developed a deep infection on the fourth day after implantation, and consequently, the neurostimulator and electrode were explanted. In addition, two patients withdrew consent to participate before the study was initiated.

In total, 73 patients (70 female) with a median age of 60 years (IQR: 50–69) completed the trial, and their results are presented. A total of 29 patients were implanted in Tromsoe, Norway, 26 patients were implanted in Aarhus, Denmark, and 18 patients were implanted in Hvidovre, Denmark. Out of the 70 female patients included, 64 had given birth to a median of 2 children (IQR: 2–3).

The aetiology of FI was obstetric anal sphincter injury in 38 patients, of whom 23 (61%) had previously undergone primary sphincter repair. At baseline, before SNM, an external anal sphincter defect was observed on ultrasound in 19 patients with a median 90° (IQR: 50–120) defect, with no significant difference between groups regarding number (G1: 13 versus G2: 6 (*p*-value 0.368) or degree of external anal sphincter (EAS) defect (*p*-value 0.380). In the remaining 35 patients, the aetiology was unknown and registered as idiopathic.

Of the 73 patients completing the study, 40 patients were randomised to G-1 and 33 patients to G-2. The baseline demographics and scores did not differ between groups (Table 2).

**Table 2:** Baseline demographics and scores for included patients and perioperative stimulation results including sensory threshold at first programming [data presented as median and (IQR)]

	Group 1	Group 2	*p*-Value
*Baseline*			
Number of patients	40	33	
Age (years)	59.5 (45.5–66)	61 (51–72)	0.148
Sex (female)	37	33	0.111
Parity	2 (1–3)	2 (2–3)	0.113
Body mass index	27.2 (23.7–33.6)	25.9 (22.6–29.3)	0.159
Idiopathic faecal incontinence	41%	55%	0.879
External anal sphincter defect < 160°	36%	26%	0.368
St. Mark’s Incontinence Score	18 (17–20)	18 (16–19.5)	0.469
VAS bowel function	17.5 (10–40)	23 (5–43)	0.851
VAS social function	40 (15–50)	50 (20–55)	0.573
VAS lifestyle	40 (10–50)	50 (25–70)	0.260
Incontinence episodes per 3 weeks	12 (6–24)	13 (9–20)	0.508
*Perioperative stimulation results and sensory threshold*			
*Motor response (mAmp):*			
Pole 0	0.8 (0.5–1.8)	1.0 (0.5–1.6)	0.418
Pole 1	1.0 (0.5–1.5)	1.0 (0.5–1.5)	0.496
Pole 2	0.5 (0.5–1.0)	0.6 (0.5–1)	0.222
Pole 3	1.0 (0.5–1.5)	1.0 (1.0–2)	0.134
Sensory threshold (volt):	0.53 (0.38–0.78)	0.60 (0.40–0.80)	0.693

### Perioperative stimulation results and sensory threshold

The perioperative inclusion criteria were met for all patients, including a motor threshold in three or more poles and at least one pole of the quadripolar lead with a motor threshold ≤ 1.5 mAmp. The mean number of poles with a motor response was 3.9 (SD 0.33). In 86% of patients, a motor response was achieved at all four poles of the quadripolar lead. The median stimulation amplitude required to achieve a motor response in any pole of the quadripolar leads were non-significantly different between G-1 or G-2 (Table 2). The ST at the initial programming the day after surgery had a median of 0.55 volts (IQR: 0.4–0.8), with non-significant differences between groups (*p*-value: 0.693) (Table 2).

### Comparisons between G-1 and G-2

Both groups improved in all evaluated parameters during the first 4 weeks after implantation (Table 3). The only statistically significant difference between the two groups was observed in the delta VAS for bowel function. In G-1 with ultra-low stimulation amplitude [0.05 V – equivalent to 9.6% (IQR: 6.5–13.4) of ST] the median improvement in the VAS for bowel function was 30 points (IQR: 10–50), significantly lower than G-2 stimulated at 90% of the ST in which a median improvement of 50 points (IQR: 10–70) was obtained (*p*-value: 0.05).

**Table 3:** Comparison between randomisation groups from week 1 to 12

Week	1–4			5–8			9–12		
	Group 1	Group 2	*p*-Value Wilcoxon rank-sum test (MW)	Group 1	Group 2	*p*-Value Wilcoxon rank-sum test (MW)	Group 1	Group 2	*p*-Value Wilcoxon rank-sum test (MW)
	0.05 volts	90% of ST		50% of ST	90% of ST		90% of ST	90% of ST	
Delta St. Mark’s Incontinence Score	4 (1–7) *n* = 35	4 (1–7) *n* = 31	0.902	4.5 (2.5–9) *n* = 36	3.5 (2–9.5) *n* = 32	0.625	5 (3–8) *n* = 38	4.5 (1–11) *n* = 30	0.853
Delta incontinence episodes	8.5 (4–21) *n* = 34	9 (5–14) *n* = 27	0.988	8 (4–19) *n* = 35	12 (6–18) *n* = 28	0.347	11 (2–20) *n* = 35	11 (6–15) *n* = 26	0.743
Reduction in FI episodes (%)	78 (54–95) *n* = 34	71 (53–92) *n* = 27	0.732	81 (60–100) *n* = 35	91 (63–100) *n* = 28	0.732	80 (61–100) *n* = 35	89 (59–100) *n* = 26	0.387
Delta VAS bowel function	30 (10–50) *n* = 34	50 (25–70) *n* = 29	0.05^*^	30 (10–60) *n* = 36	50 (10–70) *n* = 31	0.256	35 (9–55) *n* = 38	49 (25–60) *n* = 28	0.194
Delta VAS social function	20 (5–40) *n* = 33	25 (5–50) *n* = 29	0.676	20 (5–55) *n* = 35	25 (10–60) *n* = 31	0.532	30 (10–50) *n* = 37	30 (5–45) *n* = 28	0.936
Delta VAS lifestyle	25 (10–40) *n* = 33	10 (0–40) *n* = 29	0.492	28 (10–50) *n* = 35	25 (0–50) *n* = 31	0.738	30 (10–49) *n* = 37	30 (10–50) *n* = 28	0.937

Delta values: baseline – specific follow-up; data presented as median and (IQR)

*Uncorrected *p*-value: 0.0496

In the following two randomisation rounds, where the stimulation intensity was increased in G-1 to 50% of the ST (weeks 5–8) and 90% of the ST (weeks 9–12) compared with G-2 with a stable stimulation amplitude of 90% of the ST, no statistically significant difference was observed between the groups (Table 3).

### Comparison between baseline and 24-week follow-up

There was no significant difference between the groups in any evaluated parameters at the latest follow-up after 24 weeks (weeks 13–24), where all patients had been stimulated at the ST (data not shown). The median stimulation amplitude in weeks 13–24 was 0.5 volts (IQR: 0.4–0.78) in G-1 and 0.9 volts (IQR: 0.4–1.25) in G-2, with no significant differences between groups (*p*-value: 0.222). Analysed as a group, the number of incontinence episodes per 3 weeks at the latest follow-up was significantly reduced from baseline with a median of 12 episodes (IQR: 6–19) (*p*-value: < 0.001). The median reduction in incontinence episodes compared with baseline was 87.5% (IQR: 75–100). A 50% or higher reduction in incontinence episodes compared with baseline was obtained in 92% (*n* = 67) of the patients, and 33% (*n* = 24) of patients reported no FI episodes at the latest follow-up. The St. Mark’s Incontinence Score at the latest follow-up was significantly reduced from baseline with a median of 6 points (IQR: 1.5–11) (*p*-value: < 0.001).

The VAS for patient satisfaction with bowel function at the latest follow-up improved significantly from baseline, with a median of 50 points (IQR: 25–70) (*p*-value: < 0.001).

## Discussion

This randomised multicentre study is the first to demonstrate that subsensory stimulation is effective in newly implanted patients. Stimulation with 0.05 V equivalent to 9.6% (IQR: 6.5–13.4) of the ST is equally effective in reducing incontinence episodes and symptom scores as stimulation with 50–90% of the ST. The patient’s VAS for bowel function in the first period (0.05 V versus 90% of the ST) was the only score with a significantly better outcome in G-2 (90% of the ST) compared with G-1 (0.05 V) (Table 3). All other scores were not significantly different throughout the study period. This indicates that the difference may be explained by a type I error, implying that it is a false positive rather than a factual finding. The number of patients achieving this endpoint in the present trial was comparable to or even higher than those previously reported for a conventional two-stage procedure [11, 18, 19].

When the study was planned, we included a group with ultra-low stimulation intensity. The intensity was set at 0.05 V, which is the lowest possible setting, with the InterStim-II neurostimulator still being turned on. We chose a low setting because we believed it would be difficult to recruit patients for the trial with a period where the neurostimulator was turned off. We expected 0.05 V to be equivalent to 2–5% of ST on the basis of previous results [13, 20]. This stimulation intensity is expected to be too low to depolarise the afferent nerve fibres, making it effective as a placebo stimulation. All patients included were implanted in accordance with the standardised implantation technique [10]. Furthermore, a motor response (contraction) of the external anal sphincter must occur in ≤3 poles and at least one pole of the quadripolar lead, with a motor threshold ≤ 1.5 mAmp to qualify for inclusion. With these strict implantation criteria and the motor threshold, subsequently, the STs obtained at implantation were much lower than expected during the planning phase. Electrical nerve activation relies on a critical current to activate ion channels responsible for the influx of positive ions that initiate action potentials [21]. Our trial results indicate that an ultra-low stimulation intensity, equivalent to 9.6% of the ST, may be sufficient to activate these ion channels, as the efficacy of the treatment was not affected by the ultra-low amplitude stimulation. Further, electrophysiological studies should be conducted to determine the minimal electrical threshold required for nerve modulation in SNM. The efficacy that we have documented with ultra-low stimulation amplitude could theoretically be due to a methodological problem. We randomised the patients the day after implantation, and optimally, we should have introduced a washout period before starting the trial. This could eliminate the theoretical carry-over effect from the electrical stimulation used during lead placement.

The efficacy in reducing incontinence episodes was 71–91%, with no difference observed between the stimulation groups during the randomised period (Table 3). Such improvement is unlikely to be solely attributed to a placebo effect. However, the possibility of a placebo effect remains a potential confounding factor when studying functional disorders. In a randomised double-blinded trial evaluating percutaneous tibial nerve stimulation versus placebo, Knowles et al. documented that 31% of patients receiving shame stimulation experienced a 50% or higher reduction in incontinence episodes [22]. In 2020, Tan et al. published a systematic review of ten papers evaluating the placebo effect and responses following sham electrical nerve stimulation for FI and constipation [23]. They concluded that ‘Sham stimulation is associated with clinical and statistically meaningful improvements in symptoms of fecal incontinence and constipation, as well as quality of life scores, highlighting the importance of sham controls in nerve stimulation trials. Noncontrolled studies should be interpreted with caution’. Recently, Vollebregt et al. published the results from a randomised cross-over trial assessing the impact of SNM ON versus OFF (or ultra-low stimulation 0.05 V) [24]. The trial was terminated owing to a lack of recruitments, and the results were based on only 16 out of the 90 planned participants. They found that SNM conferred a non-significant reduction in faecal incontinence episodes compared with sham. The current study was designed with the expectation that 0.05 V of stimulation would be ineffective and, therefore, could be regarded as a placebo, as discussed above.

The current study confirms previous publications that subsensory stimulation is effective and that subsensory stimulations is not inferior to stimulation at or above the ST. The number of patients achieving a 50% or higher reduction in incontinence episodes in the present trial was comparable to or even higher than those previously reported [11, 13, 18, 19]. We have found that the stimulation amplitude can be further reduced from 50% below the ST to 9.6% (IQR: 6.5–13.4) of the ST without compromising treatment efficacy. Further, it is documented that subsensory stimulation is effective in patients not previously stimulated at or above the ST.

A limitation of the study is the lack of a specific sample size calculation for the randomized part of the study. A post hoc power calculation could be performed, but the results of this could be questioned. Preferably, a large multicentre randomized study exploring the effect on ultra-low stimulation amplitude versus 90% ST should be performed to confirm our positive results.

The inclusion criteria for this study were strict, and specific perioperative inclusion criteria were to be fulfilled. As a consequence of these strict criteria, the transferability of the results to daily clinical practice may not be fully integrable. However, with the standardized implantation technique [10] it was possible in all patients to achieve a motor response during lead placement in three or more poles and at least one pole of the quadripolar lead with a motor threshold ≤ 1.5 mAmp.

The timeframe of this study was extended owing to the global coronavirus disease pandemic, and our ethical approval allowed us to follow the patients only for 24 weeks. Further, research with longer follow-ups of patients stimulated at the subsensory stimulation level is needed to clarify its efficacy over time.

## Conclusions

Subsensory stimulation is feasible in newly implanted patients and an amplitude of 0.05 V equivalent to 9.6% (IQR: 6.5–13.4) of the sensory threshold is as effective in reducing incontinence episodes and symptom scores as stimulation set at 90% of the sensory threshold.

### What does this paper add to the existing literature?

This is the first paper to document that sacral neuromodulation at an ultra-low stimulation amplitude of 0.05 V is equally effective in reducing incontinence episodes and symptom scores compared with stimulation at higher amplitudes. Further, subsensory stimulation has, for the first time, been shown to be effective immediately after implantation in patients with faecal incontinence.

## Data Availability

Trial data can be assessed by request to the corresponding author.
